# D-dimer: The Risk Factor of Children's Severe Mycoplasma Pneumoniae Pneumonia

**DOI:** 10.3389/fped.2022.828437

**Published:** 2022-04-12

**Authors:** Juan Qiu, Jin Ge, Ling Cao

**Affiliations:** ^1^Children's Hospital of Capital Institute of Pediatrics, Beijing, China; ^2^Children's Hospital, Shandong University, Shandong, China; ^3^Jinan Children's Hospital, Shandong, China

**Keywords:** mycoplasma pneumoniae, pediatrics, risk factor, D-dimer, concoagulation status

## Abstract

**Objective:**

Mycoplasma Pneumoniae (MP) is an important cause of community-acquired pneumonia in children, which can cause serious consequences. There has been some research into predicting Severe Mycoplasma Pneumoniae Pneumonia (SMPP) primarily focused on pre-treatment time by macrolide, pre-hospital course, CRP and LDH et.al. while seldom reporting on concoagulation status. We designed this retrospective study to compare the difference between SMPP and Non-severe MPP (NSMPP) with an attempt to find the risk factors, with a special focus on concoagulation status.

**Method:**

We performed a retrospective study of 786 MPP patients who were hospitalized from January 1, 2016 to December 31, 2018, age ranging from 28 days to 18 years old. All patients were divided into SMPP group and NSMPP group. A univariate analysis was conducted between both groups. The factors with statistical differences were included in logistic regression analysis to summarize the predictors of SMPP. Next, the predictive value of each risk factor was calculated from the receiver operating characteristic curve (ROC curve). Patients who had D-dimer records were divided into the elevated D-dimer group (D-dimer > 308ug/L) and the control group (D-dimer ≤ 308ug/L), and the clinical manifestations were compared.

**Results:**

There was no significant difference in gender, age, pre-treatment time by macrolide, the white blood cell counts (WBC), Fibrinogen (FIB), Activated Partial Prothrombin Time (APTT), Prothrombin Time (PT) and Thrombin Time (TT) between SMPP and NSMPP. Compared with NSMPP, the pre-hospital course of SMPP was longer (*P* < 0.05), the neutrophil ratio (N%), platelet Count (PLT), C-reactive Protein (CRP), Lactate Dehydrogenase (LDH) and D-dimer were significantly higher (*P* < 0.01). The binary logistic regression analysis showed that the N%, PLT, CRP, LDH and D-dimer were the key predictors for SMPP, the N% > 67%, OR = 3.233, PLT > 445 × 10^9^ /L, OR = 2.589, LDH > 354U/L, OR = 4.335 and D-dimer level > 403 ug/L, OR = 7.316. The D-dimer possessed the best predictive value. The incidence of complications such as pleural effusion, myocardial and liver damage of MPP was higher in the elevated D-dimer group than that in the control group (*P* < 0.05).

**Conclusion:**

The N%, PLT, CRP, LDH and D-dimer were risk factors for SMPP. D-dimer was the best predictor among them. MPP patients with D-dimer > 308ug/L had more complications such as pleural effusion, myocardial and liver damage. More attention should be given in the treatment for this group.

## Introduction

Mycoplasma Pneumoniae (MP) is an important cause of community-acquired pneumonia in children, accounting for 10% to 40% of the community-acquired pneumonia in all hospitalized children (1, 2). MPP is usually a self-limiting disease, yet it can also progress into refractory or severe pneumonia. With the increasing resistance to macrolides of MP in recent years, the incidence of SMPP is also increasing. SMPP could be complicated with pleural effusion, atelectasis and necrotizing pneumonia, etc. In severe cases, respiratory failure and hypoxemia may occur, requiring mechanical ventilation, support of extracorporeal membrane oxygenation, and may even result in death (3, 4). Children with MPP can also show a wide range of extra-pulmonary manifestations (5, 6). In recent years, there have been increasing reports of MP with thrombosis, including cerebral embolism, pulmonary embolism and DIC, etc. (6), which can result in serious consequences. There has been some research into predicting SMPP mainly focused on pre-treatment time by macrolide, pre-hospital course, CRP and LDH et al. (7, 8), while seldom reporting on concoagulation status. We designed this retrospective study to compare the difference between SMPP and NSMPP with an attempt to find the risk factors, with a special focus on concoagulation status.

## Materials and Methods

### Setting and Patients

We performed a retrospective study at the Children's Hospital Affiliate to Capital Institute of Pediatrics, a tertiary referral children's hospital in Beijing, China from January 1, 2016 to December 31, 2018. The study was approved by the Research Ethics Board of this hospital.

### Diagnostic Criteria

MPP diagnosis is as follows: acute respiratory infection symptoms (fever, cough or wheezing), physical examination and chest imaging with infiltrates and laboratory confirmed MP infection that include: serum Mycoplasma pneumoniae antibody ≥ 1:320, or serum Mycoplasma pneumoniae antibody ≥ 1:160 and the MP polymerase chain reaction (PCR) positive, or MP antibody titer of recovery phase and acute phase increased or decreased by 4 times or more (9, 10).

Severe MP pneumonia that is defined as MP pneumonia with one of the following: poor general conditions, significant increase in breathing rate (RR > 70 breaths/min in infants, and 50 breaths/min in older children), Cyanosis; dyspnea, ≥ 2/3 of the lobe or multilobe involvement, pleural effusion, pulse oxygen saturation ≤ 92%, extra-pulmonary complications.

Inclusion criteria: The patients diagnosed with MPP ranging from age ≥ 28 days to 18 years old.

Exclusion criteria: (1) evidence of co-infection, including bacteria, viruses, fungi and tuberculosis, etc. (2) pre-existing chronic respiratory disease such as asthma, congenital bronchopulmonary abnormalities, bronchiectasis, etc. (3) Pre-existing other systemic diseases, such as congenital heart disease, chronic kidney disease, connective tissue disease, tumors and hematological diseases, immunodeficiency, etc.

### Data Sources

The data source for analysis of patient information was collected from electronic medical records of the hospital. The evaluation indicators include pre-treatment time by macrolide, pre-hospital course, clinical symptoms and signs, intrapulmonary and extrapulmonary complications, laboratory and imaging findings. The laboratory tests include hemoglobin, WBC counts, neutrophil ratio (N%), PLT, CRP, LDH, D-dimer (ELISA method with the normal rang <243 ug/L), FIB, APTT, PT, TT, procalcitonin (PCT), alanine aminotransferase (ALT), aspartate aminotransgerase (AST), blood urea nitrogen (BUN), blood electrolytes, creatinine kinase, MB isoenzyme (CK-MB) and cardiac troponin T (CTnT). Serum mycoplasma pneumoniae antibody and MP PCR were performed to determine whether MP infection was present. Blood, pleural effusion and nasopharyngeal aspirate/bronchoalveolar lavage fluid cultures, virus antigen detection assays (respiratory syncytial viruses, adenovirus, metapneumovirus, influenza and parainfluenza), interferon-γ release assays and T cell spot tests (T-SPOTs) for a tuberculosis infection were performed to exclude co-infection. A plain chest radiograph or chest CT was performed before or during hospitalization. An electrocardiogram (ECG), echocardiography and abdominal ultrasound were performed. If the patient was suspected to have a fungal infection, bronchoalveolar lavage fungal cultures and blood (1,3)-β-D-glucan and galactomannan detection were performed.

### Statistical Analysis

The continuous variables that followed a normal distribution were expressed as x ± s, and the non-conformities were expressed as median ± quartile. If the continuous variables of the two groups both followed a normal distribution and the variances were equal, the *T* test was used. If they did not follow a normal distribution or the variances were not equal, the Mann–Whitney U test was used. The categorical variables were analyzed by Pearson Chi-squared test. The factors with *P* < 0.05 were included in a binary logistic regression model to assess predictors for SMPP. Some continuous variables, such as neutrophil ratio, PLT counts, CRP, LDH and D-dimer levels were categorized into the 30th percentile, 60th percentile and 90th percentile. Afterwards, the accuracy of predictive factors was calculated by ROC curve. All tests were two-tailed and *P* values < 0.05 were considered significant. All statistical analyses were performed using SPSS Statistics Version 25.0.

## Results

### General Information

According to the inclusion and exclusion criteria, 786 patients were enrolled, 403 were male and 383 were female with the ratio of 1: 0.97. The age ranged from 1.3–17.4 years old, with an average age of 7.9 ± 3.0 years, of which children aged 5–9 years accounted for 56.5%, younger than 5 years old accounted for 30.3%, and 10–17 years old accounted for 13.2%.

### Clinical Characteristic Comparison Between SMPP and NSMPP

#### Univariate Analysis Between SMPP and NSMPP

According to the criteria, 540 patients were diagnosed as SMPP and 246 were diagnosed as NSMPP. There was no significant difference in gender, age, pre-treatment time by macrolid, WBC counts, neutrophil counts, FIB, APTT, PT, TT, PCT, ALT, AST and blood BUN between the two groups (*P* > 0.05). All of PCT value of the including patients were normal. Compared with NSMPP, the pre-hospital course of SMPP was longer (*P* < 0.05), the N%, PLT, CRP, LDH and D-dimer were significantly higher (*P* < 0.01) (Table 1).

**Table 1:** Clinical characteristic univariate analysis between SMPP and NSMPP.

**Variable**	**SMPP (***n*** =540)**	**NSMPP (***n*** =246)**	***P***
Age (year)	6.8 (4)	6.3 (5)	0.248
Sex (male/female)	267/273	128/118	0.713
Pre-hospital time (d)	7 (4)	7 (4)	0.036
Pre-treatment time (d)	4.6 ± 2.8	4.4 ± 2.8	0.301
WBC(×10^9^/L)	8.23 (2.94)	7.97 (3.29)	0.370
N%	0.58 (0.14)	0.54 (0.16)	0.000
PLT (×10^9^/L)	404 (160)	360 (141)	0.000
CRP (mg/L)	21 (27)	15 (22)	0.000
LDH (U/L)	326 (108.5)	299 (74.5)	0.000
D-dimer (ug/L)	332.5 (460)	216.5 (139)	0.000
FIB (g/L)	3.82 ± 0.66	3.81 ± 0.71	0.971
APTT (s)	32.3 (5.4)	32.7 (4.9)	0.113
PT (s)	11.6 (1.1)	11.5 (1.2)	0.322
TT (s)	15.1 (1.6)	15.1 (1.7)	0.308

*Data are presented as means ± SDs or median (quartile)*.

#### Binary Logistic Regression Analysis and ROC Curve Between SMPP and NSMPP

The binary logistic regression analysis was conducted for the pre-hospital course, the N%, PLT, CRP, LDH and D-dimer. It turned out that the N%, PLT, CRP, LDH and D-dimer were the predictive factors for SMPP. The N% > 67% (OR = 3.233), PLT > 445 × 10^9^/L (OR = 2.589), LDH > 354U/L (OR = 4.335) and D-dimer level > 403 ug/L (OR = 7.316) (Table 2).

**Table 2:** Binary logistic regression analysis between SMPP and NSMPP.

**Variables**	**OR**	**95%CL**	***P* value**
**N% (<55%)**	1		
55–67%	1.428	1.015–2.008	0.041
> 67%	3.233	2.186–4.784	0.00
**PLT (<348** **× 10**^**9**^ **/L)**	1		
348–445 × 10^9^ /L	1.656	1.167–2.35	0.005
> 445 × 10^9^ /L	2.589	1.779–3.767	0.000
**CRP (<12 mg/L)**	1		
12–27 mg/L	1.409	1.002–1.982	0.049
> 27 mg/L	3.491	2.355–5.174	0.000
**LDH (<295U/L)**	1		
295–354 IU/L	1.789	1.269–2,521	0.001
> 354 IU/L	4.335	2.89–6.502	0.000
**D-dimer (<219ug/L)**	1		
219–403 ug/L	1.562	1.114–2.191	0.010
>403 ug/L	7.316	4.598–11.642	0.000

**Figure 1:**
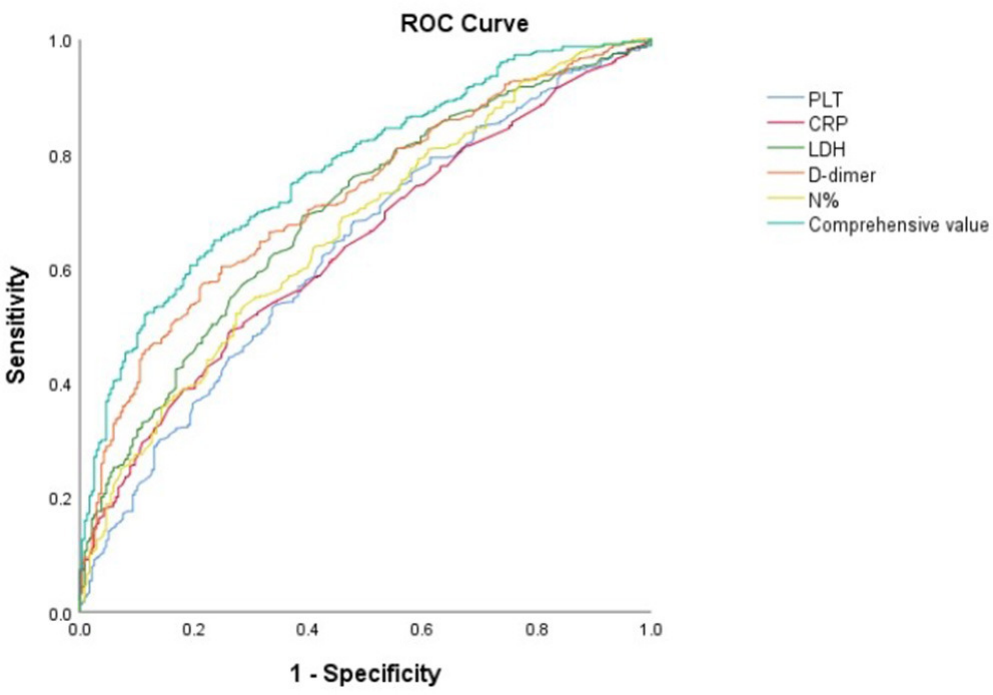
ROC curve between SMPP and NSMPP.

Subsequently, the cut-off values of the N%, PLT, CRP, LDH and D-dimer were calculated by ROC Curve as 41%, 361 × 10^9^/L, 22 mg/L, 307 U/L and 308 ug/L respectively. The D-dimer possessed the best predictive value with a sensitivity of 57.2% and specificity of 78.6%. The total predictive accuracy rate of the four factors was 77.1% (Table 3, Figure 1).

**Table 3:** Cut off value between SMPP and NSMPP.

**Variables**	**Cut-off value**	**AUC**	**Sensitivity (%)**	**Specificity (%)**
N%	**41%**	**0.663**	**0.649**	**0.765**
PLT	361 × 10^9^/L	0.631	0.682	0.521
CRP	22 mg/L	0.638	0.488	0.739
LDH	307 U/L	0.695	0.692	0.609
D-dimer	308 ug/L	0.721	0.572	0.786

#### Comparison of Complications Between MPP With Elevated D-dimer Group and Control Group

According to the above results, D-dimer possess the highest predictive value for SMPP. We divided the patients who have the D-dimer record into the elevated D-dimer group (D-dimer > 308 ug/L) and the control group (D-dimer ≤ 308 ug/L), with 54 patients without D-dimer results eliminated. The complications were compared between two groups. The results showed that in the elevated D-dimer group, the incidence of pleural effusion, myocardial and liver damage, and electrolyte disturbances was significantly higher than that in the control group, while there was no significant difference in the incidence of atelectasis, rash, anemia and otitis media between the two groups (Table 4).

**Table 4:** Complications comparison between elevated D-dimer group and control group.

**Complications**	**Elevated D-dimer Group (***n*** =333)**	**control Group (***n*** =153)**	***P*-value**
Pleural effusion	137 (41.1%)	33 (21.6%)	0.001
Atelectasis	29 (8.7%)	11 (7.2%)	0.699
Liver damage^*a*^	57 (17.1%)	3 (2.0%)	0.001
Myocardial damage^*b*^	70 (21.0%)	11 (7.2%)	0.003
Rash	30 (9.0%)	5 (3.3%)	0.079
Otitis media	6 (1.8%)	0 (0%)	0.185
Blood electrolytes disturbances	23 (6.9%)	1 (0.6%)	0.05
Anemia	23 (6.9%)	5 (3.3%)	0.281

a *Liver damage is defined as ALT or AST 2 times higher than normal value*.

b *Myocardial damage is defined as CK-MB, CTnT or ECG abnormal*.

## Discussion

It is generally believed that MPP is the most common community acquired pneumonia in school-age children. Previously, MPP was rare in infants and young children, even with the evidence of mycoplasma pneumoniae infection. A survey conducted between 2010 and 2012 showed that the incidence of MPP in 5 years and older children was higher than that in younger children (19 vs. 3%) (2). In our study, the patients under 5 years old accounted for 30.3% of all included patients, which was significantly higher than that in the study by Jain et al. (2), indicating that the incidence of MPP is trending toward a younger age range in recent years.

In the past, older children were more susceptible to severe mycoplasma pneumonia (11), while in this study, there was no significant difference in age between SMPP and NSMPP. The PCT level of all patients in severe and non-severe groups were normal, so it could not be used to diagnose MPP or determine the severity. Chan and Izumikawa observed that there was a delay in the administration of effective antibiotics in patients with SMPP, with an average delay of 9.3 days and 15 days, respectively. They believed that this might be the most important cause of fatal respiratory failure (12, 13). Some studies have also found that even if the medication was given within 3 days of onset, respiratory failure still developed. In our study, there was no difference in pre-treatment time by macrolide between SMPP and NSMPP group, so we could not conclude that the delayed use of antibiotics was a risk factor for SMPP. This might be due to the high resistance of mycoplasma pneumoniae (14), which affected the efficacy of macrolides. Neutrophils are major effectors of acute inflammation. Cacciotto et al. (15) Confirmed that Mycoplasma pneumoniae can induce the production and activation of neutrophils, thus promoting the pathogenesis of mycoplasma infection. In our study, SMPP patients have significantly higher the N% than NSMPP patients.

It is currently believed that the over-activated immune response is associated with lung injury in MPP (16, 17). When pneumonia occurs, MP and its toxins, inflammatory mediators and hypoxia can cause damage to vascular endothelium, which lead to activation, aggregation and excessive consumption of platelets. At this time, megakaryocytes in the bone marrow are activated and produce more platelets to compensate for the loss. Mirsaeidi found that the platelet counts were positively related to the length of hospital stay, the mortality and prognosis of pneumonia (18), we found that a platelet count was an independent factor for SMMP, when higher than 445 × 10^9^/L with OR = 2.589 (*P* < 0.01).

CRP was a non-specific marker of inflammation, which could rapidly increase within 4–6 h of infection. Its elevation was positively related to the degree of infection and inflammatory response (19). In our study, it is also shown that SMPP patients have higher CRP level than NSMPP patients (21 vs. 15), but not as high as an infection of bacteria.

Saraya et al. confirmed that the most significant pathological change of MPP was the accumulation of lymphocytes, neutrophils and alveolar macrophages in the area around the Bronchial Blood Vessels (PBVAs) in alveolar space (20). The continuous exudation of inflammatory cells was induced by cytokines and other inflammatory mediators (21), of which IL-8 and IL-18 played the most important role. Various studies have shown that IL-18 was directly related to the severity of MPP and could be used as a predictor of SMPP (22). Oishi and Miyashita found there was a significant correlation between serum IL-18 and LDH levels (8, 23). In this study, LDH was also found to be significantly higher in the SMMP group than in the NSMMP group and it was an independent variable used to predicte SMMP.

Studies have shown that in severe pneumonia, the interactions of inflammation and the coagulation system could aggravate lung injury. In recent years, there have been continuous reports of MPP complicated with systemic arterial and venous thrombosis, and even DIC (24). Once it occurs, it is acute and critical, with high mortality and disability rate, resulting in more serious consequences than pneumonia. D-dimer is a degradation product of cross-linked fibrin. Its increase indicates the presence of thrombosis and secondary fibrinolysis in the blood, which has an early and rapid diagnostic value for the hyper-coagulation state. Previous studies have shown that D-dimer was a predictor for 30-day mortality, the need for mechanical ventilation and circulatory support of severe pneumonia.

In our study, D-dimer in SMPP group was significantly higher than that in NSMPP group. D-dimer > 403 ug/L, the risk to become SMMP was 7 times higher than D-dimer within normal range, and its sensitivity to predicted SMPP was higher than the N%, PLT, CRP and LDH elevation. MPP with elevated D-dimer, possessed significantly more incidence of pleural effusion, myocardial and liver damage. This may reflect the mutual promotion of inflammation and coagulation, which aggravates systemic inflammation.

Some studies also found that SMPP was often accompanied by increased anti-phospholipid antibody (APA) titers, especially in children with thrombosis (25). Moreover, similar with the antiphospholipid antibody syndrome, children with MPP are prone to both venous and arterial thrombosis, suggesting that APA may be one of the mechanisms of SMPP thrombosis. Unfortunately, we did not detect APA in this study.

In summary, the N%, PLT, CRP, LDH and D-dimer were risk factors for SMPP. When D-dimer level > 403 ug/L, the patient will have more risk to be SMPP (OR = 7.316). D-dimer was the best predictor among other indicators. MPP patients with D-dimer > 308 ug/L had more complications such as pleural effusion, myocardial and liver damage. More attention should be paid to D-dimer during diagnosis. The above conclusions illustrates the important role of inflammation and hypercoagulation state in the pathogenesis of MPP.

## Data Availability Statement

The original contributions presented in the study are included in the article/supplementary material, further inquiries can be directed to the corresponding author/s.

## Ethics Statement

The studies involving human participants were reviewed and approved by 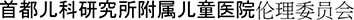. Written informed consent to participate in this study was provided by the participants' legal guardian/next of kin.

## Author Contributions

JQ, JG, and LC contributed to conception, design of the study, and wrote sections of the manuscript. JQ organized the database, performed the statistical analysis, and wrote the first draft of the manuscript. All authors contributed to manuscript revision, read, and approved the submitted version.

## Conflict of Interest

The authors declare that the research was conducted in the absence of any commercial or financial relationships that could be construed as a potential conflict of interest.

## Publisher's Note

All claims expressed in this article are solely those of the authors and do not necessarily represent those of their affiliated organizations, or those of the publisher, the editors and the reviewers. Any product that may be evaluated in this article, or claim that may be made by its manufacturer, is not guaranteed or endorsed by the publisher.
